# Persistence of Motor-Equivalent Postural Fluctuations during Bipedal Quiet Standing

**DOI:** 10.1371/journal.pone.0048312

**Published:** 2012-10-26

**Authors:** Julius Verrel, Didier Pradon, Nicolas Vuillerme

**Affiliations:** 1 Max Planck Institute for Human Development, Berlin, Germany; 2 CHU Raymond Poincaré, Garches, France; 3 AGIM Laboratory, Grenoble, France; 4 Institut Universitaire de France, Paris, France; McMaster University, Canada

## Abstract

Theoretical and empirical work indicates that the central nervous system is able to stabilize motor performance by selectively suppressing task-relevant variability (TRV), while allowing task-equivalent variability (TEV) to occur. During unperturbed bipedal standing, it has previously been observed that, for task variables such as the whole-body center of mass (CoM), TEV exceeds TRV in amplitude. However, selective control (and correction) of TRV should also lead to different temporal characteristics, with TEV exhibiting higher temporal persistence compared to TRV. The present study was specifically designed to test this prediction. Kinematics of prolonged quiet standing (5 minutes) was measured in fourteen healthy young participants, with eyes closed. Using the uncontrolled manifold analysis, postural variability in six sagittal joint angles was decomposed into TEV and TRV with respect to four task variables: (1) center of mass (CoM) position, (2) head position, (3) trunk orientation and (4) head orientation. Persistence of fluctuations within the two variability components was quantified by the time-lagged auto-correlation, with eight time lags between 1 and 128 seconds. The pattern of results differed between task variables. For three of the four task variables (CoM position, head position, trunk orientation), TEV significantly exceeded TRV over the entire 300 s-period.The autocorrelation analysis confirmed our main hypothesis for CoM position and head position: at intermediate and longer time delays, TEV exhibited higher persistence than TRV. Trunk orientation showed a similar trend, while head orientation did not show a systematic difference between TEV and TRV persistence. The combination of temporal and task-equivalent analyses in the present study allow a refined characterization of the dynamic control processes underlying the stabilization of upright standing. The results confirm the prediction, derived from computational motor control, that task-equivalent fluctuations for specific task variables show higher temporal persistence compared to task-relevant fluctuations.

## Introduction

Both theoretical and empirical evidence indicates that the central nervous system is able to exploit motor equivalence (the abundance of biomechanical degrees of freedom, DOF, over task variables) in order to stabilize motor performance [1]–[4]. This could be achieved by a biological control scheme selectively suppressing task-relevant variability (TRV) while allowing task-equivalent variability (TEV) to occur. Motor-equivalent stabilization has mainly been studied by comparing the *amount* of TEV and TRV [5]. In addition, selective control of task-relevant deviations, as proposed by dynamic models of multi-DOF coordination [4], [6], [7], should also lead to different temporal structures of TEV and TRV. More specifically, as such a control scheme exerts tight control on TRV while allowing TEV to accumulate (within functional constraints), TEV should exhibit higher temporal persistence compared to TRV. The present study was specifically designed to test this prediction for the sensorimotor task of unperturbed bipedal standing.

Previous work on prolonged bipedal standing provides empirical evidence that postural fluctuations are structured (geometrically) in a way that stabilizes upright posture during unperturbed stance [8]. Hsu et al [8] analyzed sagittal joint angle data from six joints using the uncontrolled manifold (UCM) approach [3]. In the UCM analysis, a geometric forward model (mapping from elemental variables, such as joint angles, to a task variable, such as the center of mass, CoM) is used to define the submanifold (the UCM) of the space of elemental variables (joint angles) which leave the specific task variable invariant. According to the UCM hypothesis [3], stabilization of a task variable is achieved by selectively controlling and correcting joint angle deviations from the task-equivalent manifold (the UCM). To analyze this quantitatively, variability in elemental variables is decomposed into components parallel and orthogonal to the UCM, representing TEV and TRV, respectively. In the abovementioned study [8], postural fluctuations were decomposed into TRV and TEV with respect to three task variables in the sagittal plane: (1) center of mass (CoM) position, (2) head position, and (3) head orientation (pitch). For all three variables, results showed that the amplitude of TEV exceeded the amplitude of TRV, indicating motor-equivalent stabilization. This was the case both with and without visual feedback. Moreover, the UCM index (ratio between TEV and TRV) was higher for CoM and head position compared to head orientation, suggesting that head orientation was stabilized to a lesser extent than the other two task variables.

The temporal structure of postural control has mostly been analyzed by applying linear and non-linear methods to univariate center of pressure time series [9]–[11]. The analysis of upright posture in terms of center of foot pressure profiles implicitly or explicitly assumes that the human body in bipedal upright stance can appropriately be modeled as a single inverted pendulum [12] and thereby neglects the multivariate nature of human postural control [8], [13]–[15]. According to computational principles of multi-DOF coordination [2]–[4], [7], including postural balance control [16], the central nervous system may control important aspects of a motor task by selectively constraining variability to task-equivalent subspaces. As stated above, the UCM hypothesis suggests that control is small or absent along the task-equivalent subspace (the UCM), while deviations orthogonal to the UCM are corrected in order to ensure successful task performance. Similarly, the minimal intervention principle [4], [6] states that movement variability “is not eliminated, but instead is allowed to accumulate in task-irrelevant dimensions”. Accordingly, task-equivalent fluctuations are not only expected to be larger in amplitude [8], but should also exhibit stronger temporal “persistence” (i.e., a tendency to accumulate over time) compared to task-relevant fluctuations. This hypothesis is supported by evidence from manual pointing [17] as well as the coordination between step length and step duration during treadmill walking [18]. However, to our knowledge, this question has not been investigated with respect to whole-body fluctuations in a continuous task such as unperturbed bipedal standing yet. Due to neuromechanical delays in sensorimotor control processes [19] and the hypothesized accumulation of fluctuations over time, evidence for motor-equivalent stabilization of quiet standing is expected to be found primarily at intermediate and longer time scales.

The present study aims at clarifying the relation between motor-equivalent and temporal structure of postural fluctuations during prolonged bipedal quiet standing without visual feedback. Kinematic postural data of 5 minutes of unperturbed bipedal standing were decomposed in to TEV and TRV with respect to four hypothetical task variables: (1) center of mass (CoM) position, (2) head position, (3) trunk orientation, and (4) head orientation. These variables were chosen based on their role for postural equilibrium and orientation [20], and to allow direct comparison with results from a previous study on motor-equivalent stabilization of upright balance [8]. Persistence of whole-body postural fluctuations was characterized by the time-lagged autocorrelation. Based on the reasoning presented above, we predicted that TEV would not only exceed TRV [3], [8], but also exhibit higher temporal persistence for some or all of the task variables.

## Results

We analyzed the motor-equivalent structure of prolonged (five minutes) bipedal upright standing. A 6-DOF sagittal plane model was used to describe postural fluctuations in terms of joint angles. Based the uncontrolled manifold (UCM) [3] and the covariation by randomization method (COV) [21] method, the (geometric) motor-equivalent structure of postural fluctuations was characterized with respect to four hypothesized task variables previously described in the literature [8], [20]: anterior-posterior CoM position, head position, trunk orientation and head orientation. Based on previous work, we expected to find evidence for motor-equivalent stabilization of these variables. For the UCM analysis, this would be reflected in greater amounts of task-equivalent (TEV) compared to task-relevant variability (TRV), or, equivalently, a UCM index (ratio TEV/TRV) greater than 1.

**Figure 1:**
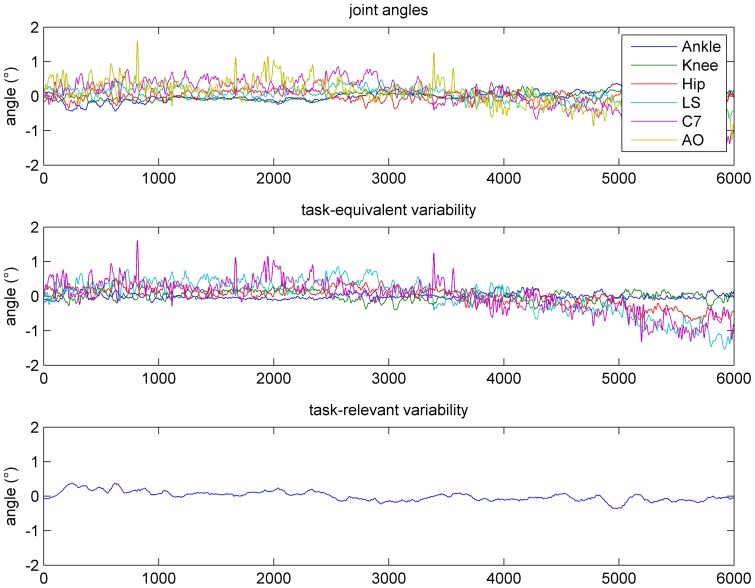
Mean-centered sample time series (60s) for joint angles (A), task-equvialent fluctuations w.r.t. CoM (B), and task-relevant fluctiations w.r.t. CoM (C).

Our main research questions concerned the temporal structure of TEV and TRV. Based on computational principles of motor control [2], [4], we predicted that TEV would show greater temporal persistence (quantified by the dimension-wise autocorrelation) compared to TRV. Sample data for joint angles, TEV and TRV (both with respect to CoM position) are shown in Figure 1.

### Amount of variability in joint angles

**Figure 2:**
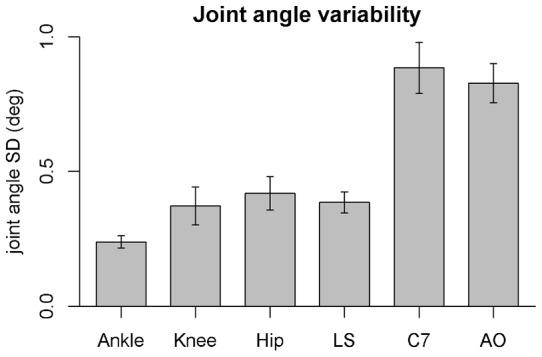
Variability(SD) for each of the six sagittal joint angles. Error bars represent SEM.

Joint angle variability (Figure 2) varied significantly across joint angles [F(5, 65) = 18.0, p<0.001, η^2^ = 0.52]. Pairwise comparisons showed that variability was significantly higher in C7 and AO than in the other four joints (ankle, knee, hip and LS) [all p<0.001].

### UCM and COV indices

**Figure 3:**
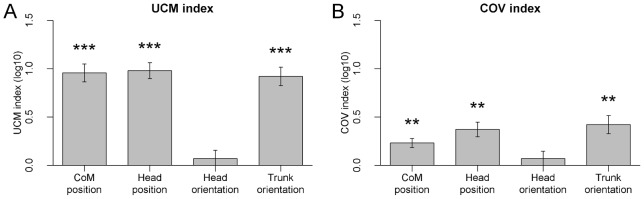
Log-transformed UCM and COV indices for the four task variables. Significant deviations from 0 are indicated (* p<0.05, ** p<0.01, *** p<0.001, Bonferroni-Holm corrected). Error bars represent SEM.

Significant UCM effects (log-transformed UCM index greater than 0, Figure 3A) were observed for CoM position, head position and trunk orientation [t(13)>9, p<0.001], but not for head orientation [t(13) = 0.85, p = 0.41]. UCM indices differed between task variables [F(3,39) = 31.5, p<0.001, η^2^ = 0.59], with head orientation having a smaller UCM index compared to the other three variables [all p<0.001].

The COV index (Figure 3B) showed a similar pattern, with significant COV effects for all task variables [t(13)>4, p<0.002], except for head orientation [t(13) = 0.96, p = 0.35]. COV indices differed between task variables [F(3,39) = 7.37, p<0.001, η^2^ = 0.20], with head orientation having a smaller COV index compared to trunk orientation and head position [both p<0.05].

Thus, both the UCM and COV analysis indicate that the geometric structure of postural fluctuations was consistent with motor-equivalent stabilization of CoM position, head position and trunk orientation.

### Persistence in joint angles and task variables

**Figure 4:**
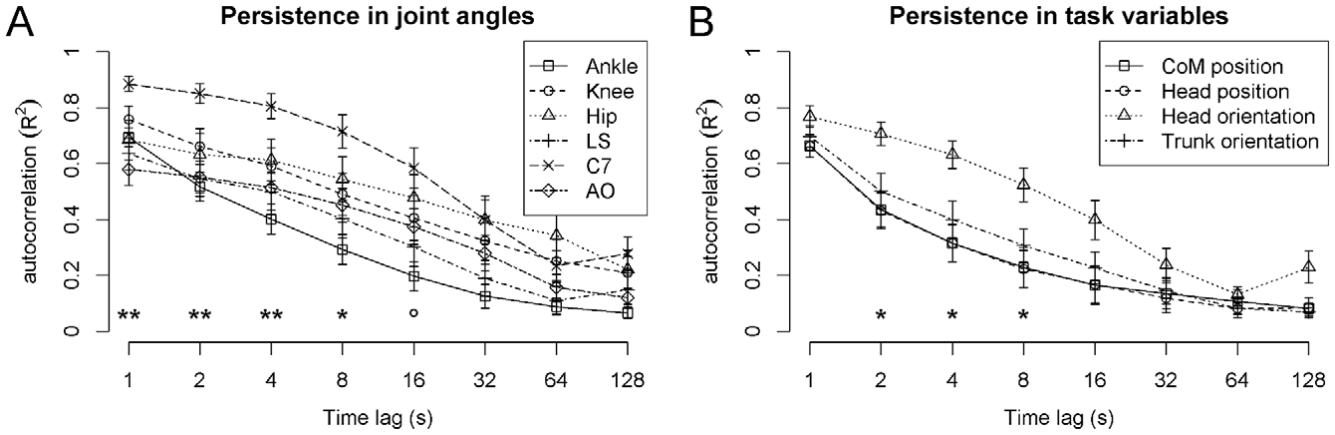
Persistence(lagged-autocorrelation) of joint angles and task variables. Trends and significant simple effects of joint angle (task variable) at each time lag are indicated (° p<0.1, * p<0.05, ** p<0.01, *** p<0.001, Bonferroni-Holm corrected). Error bars represent SEM.

Persistence in joint angles and task variables at different time lags is shown in Figure 4. For the joint angles, the omnibus ANOVA showed significant main effects of Joint Angle [F(5,65) = 2.83, p<0.05, η^2^ = 0.12] and Time Lag [F(7,91) = 104.0, p<0.001, η^2^ = 0.42], and a significant interaction between Joint Angle and Time Lag [F(35,455) = 1.65, p<0.05, η^2^ = 0.03]. As indicated in Figure 4A, pairwise comparisons showed significant differences between Ankle and C7 (at 2 s, 4 s, and 8 s), LS and C7 (at 1 s, 2 s, and 4 s), and between C7 and AO (at 1 s and 2 s).

Persistence in task variables showed significant main effects of Task Variable [F(3,39) = 4.46, p<0.015, η^2^ = 0.15] and Time Lag [F(7,91) = 73.1, p<0.001, η^2^ = 0.50], and no significant interaction between Task Variable and Time Lag [F(21,273) = 1.06, p>0.1]. As indicated in Figure 4B, comparison of the means suggests that persistence of head orientation was higher compared to that of the other three variables at lags 2,4, and 8 seconds. However, none of the pairwise comparisons was significant (all p>0.1).

### Persistence of task-equivalent (TEV) and task-relevant (TRV) fluctuations

**Figure 5:**
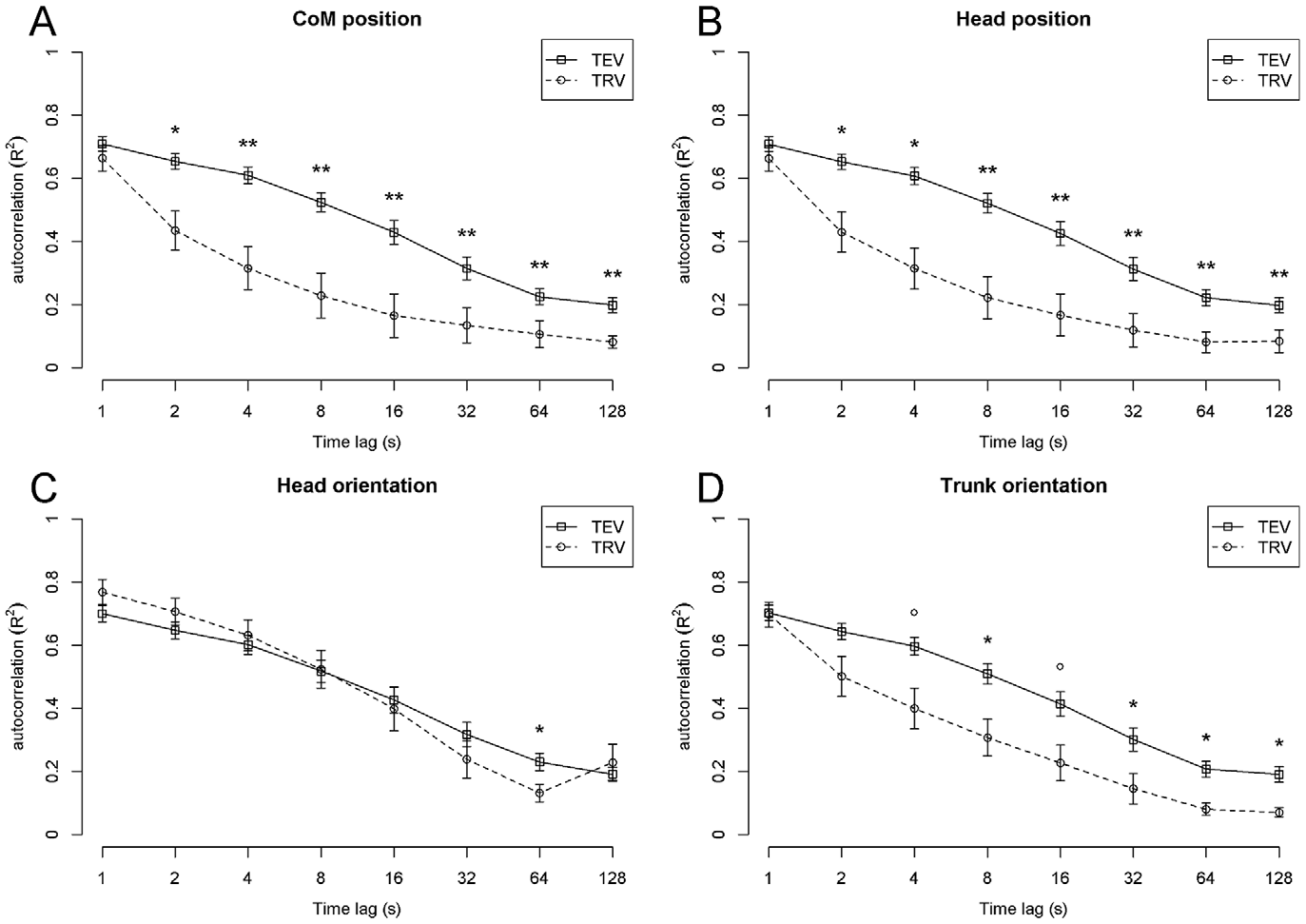
Persistence(lagged-autocorrelation) of TEV and TRV for each of the four task variables. Trends and significant differences between TEV and TRV at each time lag are indicated (° p<0.1, * p<0.05, ** p<0.01, *** p<0.001, Bonferroni-Holm corrected). Error bars represent SEM.

Figure 5 shows persistence in TEV and TRV, separately for each of the four task variables. Results of the ANOVAs assessing the effects of Variability Component (TEV vs. TRV) and Time Lag are reported below.

For CoM position, significant main effects of Variability Component [F(1,13) = 19.4, p<0.001, η^2^ = 0.29], Time Lag [F(7,91) = 55.1, p<0.001, η^2^ = 0.43], and an interaction between Variability Component and Time Lag [F(7,91) = 7.62, p<0.001, η^2^ = 0.08] were found.

Comparable effects were found for head position: significant main effects of Variability Component [F(1,13) = 21.4, p<0.001, η^2^ = 0.32] and Time Lag [F(7,91) = 50.1, p<0.001, η^2^ = 0.44], and an interaction between Variability Component and Time Lag [F(7,91) = 6.68, p<0.001, η^2^ = 0.10]; and for trunk orientation: main effects of Variability Component [F(1,13) = 10.5, p<0.01, η^2^ = 0.20] and Time Lag [F(7,91) = 59.1, p<0.001, η^2^ = 0.48], and a significant interaction between Variability Component and Time Lag [F(7,91) = 5.61, p<0.001, η^2^ = 0.07]. The ANOVA for head orientation showed a significant main effect of Time Lag [F(7,91) = 48.9, p<0.001, η^2^ = 0.49] and a significant interaction between Variability Component and Time Lag [F(7,91) = 5.45, p<0.001, η^2^ = 0.06].

Significant differences between TEV and TRV at each time lag are indicated in Figure 5. Both CoM position and head position showed higher persistence for TEV than TRV across a wide range of time lags (2–128 s). A similar pattern, though less pronounced, was found for trunk orientation, with TEV-persistence being significantly larger than TRV-persistence at time lags of 8 s, 32 s, 64 s, and 128 s (and corresponding trends at 4 s and 16 s). For head orientation, such an effect was only found at one time lag (64 s).

In order to control for potential artifacts due to differences in task-sensitivity of the six joint angles, the persistence analysis of TEV and TRV was also performed with dimension-wise autocorrelations weighted according to task sensitivity (as determined from the Jacobian). For CoM position and head position, the pattern of results was similar to the one with equally weighted autocorrelations. In contrast, head and trunk orientation showed no significant difference in persistence between TEV and TRV at any time lag.

To summarize, for CoM position and head position, and (to a lesser extent) trunk orientation, persistence of postural fluctuations was higher in TEV than in TRV, confirming the main prediction of the present study for these task variables. In contrast, head orientation did not show any systematic difference (across time scales) in the persistence of TEV versus TRV.

## Discussion

The present study was designed to test the prediction that, during prolonged unperturbed bipedal standing, whole-body postural fluctuations show higher temporal persistence in multivariate components which do not affect functionally relevant task variables (TEV), compared to task-relevant components (TRV). This prediction was confirmed for three of the four investigated task variables: CoM position, head position, and trunk orientation (though to a lesser extent). These three task variables are also the ones for which the original UCM and COV analysis indicated that motor-equivalence was exploited to minimize task variability (i.e., UCM and COV indices significantly larger than 1). In contrast, no analogous effects (neither in the traditional UCM/COV analysis, nor regarding persistence of TEV versus TRV) were observed for the fourth task variable we analyzed, head orientation. As the autocorrelation of a time series is mathematically independent of its variance, the two approaches provided independent and convergent evidence about the presence or absence for motor-equivalent stabilization of functional task variables.

### Temporal structure of task-equivalent fluctuations

By combining analyses of the temporal and task-equivalent variability structure, the present study allows a refined characterization of the dynamic control processes underlying the stabilization of upright standing. While our current analysis does not allow direct quantitative comparisons with computational models of multi-DOF coordination [2], [4], [7], the qualitative pattern of results is in line with the abovementioned models. Both the UCM framework and optimal feedback control entail a “minimal intervention principle”, according to which deviations from a planned state (for instance a bipedal posture) are only corrected when they interfere with task performance [4], [6]. Such a control principle should not only lead to larger *amplitude,* but also to greater *persistence* (i.e., accumulation over time) of task-equivalent fluctuations, as found in the present study.

Differences in persistence of TEV and TRV emerged only at intermediate and longer time lags (at least 2 s). This effect of time scale may be a consequence of the inertial properties of the musculoskeletal system, neuromuscular delays, as well as different kinds of control processes (open-loop versus closed loop control) operating at different time scales (e.g., below vs. above 1 Hz) during unperturbed bipedal standing [22]. That is, the effects of postural corrections, which are hypothesized to deal differentially with task-equivalent and task-relevant deviations, may only become visible at time lags of two seconds and above.

### Differences between task variables

The pattern of results, both with respect to amount and persistence of postural variability, differed between the four task variables. As CoM position is directly related to whole-body equilibrium during bipedal quiet standing, the observed task-equivalent stabilization according to both the geometric and the temporal analyses makes sense from a functional point of view. On the other hand, postural orientation involves the alignment of body parts (in particular the trunk and the head) with respect to gravity, support surface and visual environment [20]. While the role of postural orientation seems less important compared to body equilibrium, experimental evidence indicates that trunk and head orientation provide important reference frames for both perception and action and are actively stabilized during bipedal quiet standing [20], [23]–[26]. According to our results, both head position (which may be seen as a global indicator of whole-body orientation) and trunk orientation were indeed stabilized in a task-equivalent way. In contrast, no systematic stabilization was found for head orientation in the present study, which may be explained by the absence of a visual component in the postural task (eyes closed, no visual fixation). The difference to a previous study [8], which did find evidence for motor-equivalent stabilization of head orientation even in trials without visual feedback (though to a lesser extent than CoM and head position), could be partly due to the fact that the study by Hsu et al [8] included trials with instructed visual fixation (i.e., subjects were instructed to look straight ahead and focus their attention on a scenic picture that was hung 3-m in front of them), which may have primed participants to stabilize their head even in closed eyes trials.

### Methodological considerations

In the present study, we quantified persistence by the averaged coefficient of determination (squared autocorrelation) at different time lags. In developing the analysis, alternative measures of persistence were considered, in particular 1) the univariate autocorrelation of the first principal component, and 2) the multivariate coefficient of determination (using multivariate autoregression). For these two measures, we also found reliable differences between TEV and TRV as well (which were actually more pronounced than the present results), but could not exclude biases due to differences in dimensionality of TEV and TRV. Therefore, using the averaged cross-correlation appeared as the soundest and most conservative choice.

The instruction to remain as still as possible, used in the present study, may lead subjects to suppress naturally occurring fluctuations. On the one hand, this may limit generalizability of our results to everyday postural behavior. On the other hand, however, previous results having reported that the wording of the instructions given to the participants significantly influenced the outcome of traditional static posturography [27]–[30], and should hence be considered in the standardisation of the posturographic protocols. In the present study, the instruction to “remain as still as possible” was chosen in order to minimize superfluous body motion, such as postural changes that may have artificially increased the temporal persistence of postural fluctuations. In this sense, the fact that reliable differences both in amount and temporal persistence were found between TEV and TRV even under this relatively constraining instruction may also be considered as a strength of the present results.

Postural fluctuations in the present experiment were small (SD below 1° for some joint angles in some participants), raising the question to what extent measurement inaccuracies may have influenced the present results. To test for this possibility, we performed the same analysis after adding Gaussian noise (uncorrelated, normally distributed, SD = 2mm) to the raw kinematic data. Importantly, the pattern of results was the same for data with added noise, both with respect to the original UCM and COV analyses and with respect to differences in persistence between TEV and TRV.

Finally, body postures were represented in terms of joint angles in the present study, as done in most studies on multi-DOF coordination we are aware of. However, segment elevation angles are also sometimes used in biomechanics research, and there is evidence showing that the choice of coordinate system may influence the results of UCM and COV analysis [31]. To test for this possibility, we re-analyzed the data using segment elevation angles. As two of the task variables are segment elevation angles themselves (head and trunk orientation), this analysis only made sense for the two remaining task variables: CoM and head position. The results of this analysis are very similar to the analysis based on joint angles. In particular, both task variables showed significant UCM and COV effects, and for both variables, temporal persistence was larger for TEV than for TRV for time lags 2–128 s (CoM position) and 1–128 s (head position). Thus, at least for the present study, the choice of coordinates (joint angles vs. segment angles) does not seem to strongly influence the results.

### Conclusion and Outlook

By combining geometric and temporal analyses of postural fluctuations, the present analysis allowed testing qualitative predictions from computational models of multi-DOF coordination (“minimal intervention principle”) concerning the temporal structure (persistence) of motor-equivalent fluctuations. The prediction was confirmed for three of the four task variables under consideration, in particular for the CoM position, which is the task variable most directly linked to whole-body equilibrium. Future research should determine how task constraints (e.g., presence or absence of visual feedback, nature of the support) and suprapostural tasks may influence the persistence of motor-equivalent fluctuations for different task variables. Moreover, if stabilization of upright posture under unperturbed and perturbed conditions depends on shared mechanisms, as has been proposed based on analyses of center of pressure fluctuations [32], it would also be of interest to analyze potential relationships between the temporal structure of motor-equivalent fluctuations during quiet standing (as in the present study) and in response to external perturbations [33].

## Materials and Methods

### Participants

Fifteen healthy young adults [age: 21±1.8 years (mean ± SD), 10 men 5 women, body weight: 66.9±10.5 kg] voluntarily took part in the study. The study was conducted in accordance with the Declaration of Helsinki and approved by the ethics committee of the Société Française des Technologies pour l′ Autonomie et de Gérontechnologies. Participants gave their informed written consent to the experimental procedure. The data of one participant had to be excluded due to head motion in the horizontal plane (yaw), introducing excessive error for the sagittal plane model (see below).

### Experimental setup and procedures

An optoelectronic motion capture system with 8 cameras (1.4 M pixels, Motion Analysis Corporation, CA, USA, Sampling Frequency 100 Hz, spatial accuracy: 2 mm) was used to record whole-body kinematics. The trajectories of 36 markers placed on anatomical landmarks according to the Helen Hayes marker set [34], modified by adding five markers: C7, right and left ear, fifth metatarsal on the foot. The same investigator placed the markers at all sessions. Participants stood with closed eyes, feet parallel and separated by 10 cm, arms hanging freely by the sides of the body, and were instructed to remain as still as possible [27].

### Data analysis

Data were analyzed in Matlab (R2011b, The MathWorks, Inc.), using custom-written software. Raw kinematic data were lowpass filtered with a bidirectional Butterworth filter of order 5 and with a cut-off frequency of 10 Hz [8]. Hip joint centers were estimated using linear regression as in the conventional gait model [35]. Position data were averaged between symmetric markers (e.g., left and right ankles). A 6-DOF sagittal plane model was defined, taking the toe, ankle, knee, hip joint, sacral, C7 and head markers/positions as joint centers, as in a previous study on motor-equivalent stabilization of prolonged standing [8]. Based on this model, joint angle time series (planar angles) were computed for the following six angles: ankle, knee, hip, lumbo-sacral (LS), lower neck (C7), and atlanto-occipital (AO).

The UCM approach [3] was used to decompose joint angle variability data into task-equivalent and task-relevant components (TEV, TRV), with respect to four task variables: (1) anterior-posterior CoM position, (2) anterior-posterior head position, (3) trunk orientation (pitch), and (4) head orientation (pitch). This was achieved by defining forward models, mapping joint angle configurations to each of the task variables. The whole-body CoM position was estimated using published relative segmental masses and CoM positions [36]. A local linear approximation of the forward model (its multivariate derivative, represented by the Jacobian matrix) is used to split the 6-dimensional joint angle space into a 5-dimensional task-equivalent subspace (the null space of the Jacobian) and a 1-dimensional task-relevant subspace (the range space of the Jacobian). The task sensitivities (Jacobian coefficients) are indicated in Table 1. For further details, we refer to previous studies applying the UCM analysis in the context of unperturbed bipedal stance [8], [15]. Variability in task-equivalent and task-relevant subspaces (TEV, TRV) was normalized to the dimensionality of the subspaces (5 and 1, respectively). A task variable is considered to be stabilized by motor-equivalent coordination when the UCM index, defined as the ratio TEV/TRV, reliable exceeds 1 (or, equivalently, TEV>TRV).

**Table 1:** Task sensitivities (coefficients of the Jacobian, mean and SD) of the six joint angles for the different task variables.

Task variable	Ankle	Knee	Hip	LS	C7	AO
CoM position (mm/°)	15.79 (1.05)	9.09 (0.49)	3.48 (0.17)	2.08 (0.12)	0.20 (0.02)	0 (0)
head position (mm/°)	26.6 (1.53)	19.43 (0.92)	12.35 (0.56)	10.05 (0.51)	2.51 (0.21)	0 (0)
head orientation (d.u.)	1 (0)	1 (0)	1 (0)	1 (0)	1 (0)	1 (0)
trunk orientation (d.u.)	1 (0)	1 (0)	1 (0)	1 (0)	0 (0)	0 (0)

In the original UCM analysis [3], variance in all joint angles is weighted equally in the computation of TEV and TRV. This may lead to an artificial inflation of UCM indices when high variability occurs in joint angles with low task-sensitivity, that is, joint angles that have little influence on the task variable currently analyzed [37]–[39].

To control for this potential effect, a complementary approach was used: the covariation by randomization (COV) method [21], which assesses the use of motor-equivalence by projecting original and decorrelated data into task space and comparing variability at that level. Similarly to the UCM analysis, a COV index is defined by the ratio in task variability of decorrelated and original data, and the control hypothesis is confirmed when the COV index reliably exceeds 1 [for details, see 39]. Persistence of the two variability components (TEV, TRV) was quantified by the time-lagged squared auto-correlation coefficient (R^2^, i.e., the coefficient of determination), at time lags of 1,2,4,8, 16, 32, 64, and 128 seconds. For TEV, which is a 5-dimensional time series, the squared auto-correlation was computed separately for each dimension and averaged across dimensions. Analogously to the discussion of UCM and COV analyses above, the definition of TEV may also lead to biases in the auto-correlation analysis, when joints with low task sensitivity exhibit high temporal persistence. The task sensitivities for the different task variables are indicated in Table 1. To control for the potential influence of this effect, the multivariate autocorrelation was computed in two ways: by taking the (equally weighted) average as well as by weighting the univariate autocorrelations according to the squared task sensitivity (as defined by the Jacobian coefficients) of the corresponding joints. Thus, using the weighted average reduces the contribution of joint angles that have little influence on the task variable under consideration.

### Statistical analysis

Statistical analysis was performed in R [40], [41]. To correct for non-normal distribution, UCM and COV indices were log-transformed [42], and persistence measures (coefficients of determination) were transformed using the inverse hyperbolic tangent (with definition interval rescaled to [0,1]).

The distribution of variability across joint angles was analyzed by a univariate analysis of variance (ANOVA) with within-subject factor Joint Angle, as well as pairwise comparisons (t-tests) between joint angles. For the UCM and COV indices, one-sample t-tests were used to assess if they differed significantly from 1 (i.e., whether the log-transformed values differed from 0). In addition, differences of these indices between task variables were assessed using a univariate ANOVA with within-subject factor Task Variable, and corresponding paired t-tests.

Persistence of joint angles (task variables) was analyzed by omnibus ANOVAs with within-subject factors Joint Angle (Task Variable) and Time Lag (8 levels, 1 s – 128 s), as well as separately at each time lag, by means of a univariate ANOVAs and paired t-tests.

Finally, persistence of TEV and TRV was compared, separately for each task variable, by ANOVAs with within-subject factors Variability Component (TEV, TRV) and Time Lag (8 levels, 1 s −128 s). In addition, differences between TEV and TRV were also assessed by paired t-tests at each time lag.

The threshold for statistical significance of ANOVAs was set at 0.05. Sphericity corrections (Greenhouse-Geisser) were applied where necessary. Effect sizes are reported as generalized eta-squared [43]. For simple effect analyses and t-tests, the significance level was Bonferroni-Holm corrected [44].
